# Influence of the Time of Day and Fasting Duration on Glucose Level following a 1-Hour, 50-Gram Glucose Challenge Test in Pregnant Women

**DOI:** 10.1371/journal.pone.0112526

**Published:** 2014-11-13

**Authors:** Panchalli Wang, Mei-Chun Lu, Cheng-Wei Yu, Yuan-Horng Yan

**Affiliations:** 1 Department of Obstetrics and Gynecology, Ditmanson Medical Foundation Chia-Yi Christian Hospital, Chia-Yi City, Taiwan; 2 Department of Medical Research, Ditmanson Medical Foundation Chia-Yi Christian Hospital, Chia-Yi City, Taiwan; 3 Department of Nutrition, Ditmanson Medical Foundation Chia-Yi Christian Hospital, Chia-Yi City, Taiwan; 4 Department of Internal Medicine, Ditmanson Medical Foundation Chia-Yi Christian Hospital, Chia-Yi City, Taiwan; 5 Institute of Occupational Medicine and Industrial Hygiene, College of Public Health, National Taiwan University, Taipei, Taiwan; Rajarata Univeresity of Sri Lanka, Sri Lanka

## Abstract

**Background:**

Previous studies have shown that the time of day (TD) of glucose measurement and the fasting duration (FD) influence the glucose levels in adults. Few studies have examined the effects of the TD and FD on the glucose level following a 1-hour, 50-gram glucose challenge test (GCT) in pregnant women in screening for or diagnosing gestational diabetes mellitus (GDM). The objective of this study was to investigate the influence of the TD (morning, afternoon, night) and the FD (the time of the last food ingestion as follows: ≤1 hour, 1–2 hours, and >2 hours) by examining their combined effects on the glucose levels following a 50-gram GCT in pregnant women.

**Methods and Results:**

We analyzed the data of 1,454 non-diabetic pregnant Taiwanese women in a prospective study. Multiple linear regression and multiple logistic regression were used to estimate the relationships between the 9 TD-FD groups and the continuous and binary glucose levels (cut-off at 140 mg/dL) following a 50-gram GCT, after adjusting for maternal age, nulliparity, pre-pregnancy body mass index, and weight gain. Different TD and FD groups were associated with variable glucose responses to the 50-gram GCT, some of which were significant. The estimate coefficients (β) of the TD-FD groups “night, ≤1 hr” and “night, 1–2 hr” revealed significantly lower glucose concentrations [β (95% confidence interval [CI]): −6.46 (−12.53, −0.38) and −6.85 (−12.50, −1.20)] compared with the “morning, >2 hr” group. The TD-FD groups “afternoon, ≤1 hr” and “afternoon, 1–2 hr” showed significantly lower odds ratios (OR) of a positive GCT; the adjusted ORs (95% CI) were 0.54 (0.31–0.95) and 0.58 (0.35–0.96), respectively.

**Conclusions:**

Our findings demonstrate the importance of standardizing the TD and FD for the 1-hour, 50-gram GCT. In screening for and diagnosing GDM, the TD and FD are modifiable factors that should be considered in clinical practice and epidemiological studies.

## Introduction

Previous studies have shown that the time of day (TD) of glucose measurement and the fasting duration (FD) influence the glucose level in adults [1]–[4]. Studies in pregnant women have shown the effects of the TD (or circadian or diurnal variations) on the results of a gestational 1-hour, 50-gram glucose challenge test (GCT) [5]–[8]. Previous studies have demonstrated that the fasting duration (FD) (or the time since the last caloric intake) might influence the gestational GCT [6], [9]. Few studies have investigated the effects of the TD and the FD on the glucose levels following gestational GCT in pregnant women [6].

The two-step approach for the diagnosis of gestational diabetes mellitus (GDM), which consists of a 1-hour, 50-gram GCT combined with a 100-gram oral glucose tolerance test (OGTT), is supported by the American College of Obstetricians and Gynecologists (ACOG); it has been widely used in nearly all the hospitals in Taiwan for more than 10 years. In Taiwan, the general recommendation is that all pregnant women complete the 1-hour, 50-gram GCT, which is covered by the National Health Insurance (NHI) policy. The 1-hour, 50-gram GCT portion of the current two-step approach for the diagnosis of GDM does not specify the parameters for the TD or the FD, and the instructions regarding whether pregnant women should fast for this test are inconsistent. Appointment schedules for outpatient services in Taiwan are very busy and include morning, afternoon, and night appointments in clinics where pregnant women can undergo the 1-hour, 50-gram GCT.

Recently, based on the Hyperglycemia and Adverse Pregnancy Outcome (HAPO) study, the International Association of Diabetes and Pregnancy Study Groups [10] presented new consensus guidelines for the diagnosis of GDM using a 1-step, 2-hour, 75-gram OGTT [10], [11]. The HAPO study and the IADPSG guidelines do not consider the TD or FD. To our knowledge, no study has investigated the associations between the TD, FD, and glucose measurements with the 2-hour, 75-gram OGTT. The 1-hour, 50-gram GCT and the 2-hour, 75-gram OGTT are glucose tolerance tests, and they should be used carefully in pregnant women [12]. Understanding the modifiable factors that might cause variations in glucose tolerance test results is important. Thus, the objective of this study was to investigate the influence of the TD and FD on glucose levels following a 1-hour, 50-gram GCT in pregnant Taiwanese women.

## Materials and Methods

### Study participants

This hospital-based prospective study was conducted in a 1000-bed teaching hospital in southern Taiwan. During the study period, March - October 2011, all patients who underwent a 50-gram GCT at 24–28 weeks of gestation at the obstetric clinic at the Ditmanson Medical Foundation Chia-Yi Christian Hospital (DMF-CYCH) were enrolled. The patients with pre-pregnancy diabetes, multi-fetal pregnancies, and a history of GDM were excluded. The DMF-CYCH institutional review board (IRB) approved this study (CYCH-IRB No. 100030), and all the study participants provided written informed consent to participate in the study.

### Measurements of the TD, FD and glucose level

The venous plasma glucose levels were measured by the glucose oxidase method using a Hitachi 7170 automatic analyzer (Hitachi Co., Tokyo, Japan) in the DMF-CYCH central laboratory, according to a standard clinical protocol. The TD was defined as the time of the blood draw minus one hour; the times of all blood draws were obtained from the same computer in the DMF-CYCH central laboratory. Nurses from the Department of Community Health at DMF-CYCH administered a face-to-face interview questionnaire to the patients to collect basic data (including maternal age, parity, pre-pregnancy body mass index [BMI], and weight gain) and FD data.

### Statistics

The patients were stratified into 9 groups, designated as the TD-FD groups, by the TD (morning: 9 AM–12 PM, afternoon: 12–5 PM, and night: 5–10 PM) and FD (the time elapsed from the patient's last food ingestion prior to the GCT: ≤1 hr, 1–2 hr, and >2 hr). The chi-square test was used to analyze the distribution of the known risk factors for the glucose levels in each TD-FD group. The known risk factors included maternal age, nulliparity, pre-pregnancy BMI, and weight gain. BMI was calculated as weight [3]/[height (m)]^2^ and was categorized according to the criteria of the Bureau of Health Promotion of the Department of Health in Taiwan. Weight gain was defined as the percentage of gestational weight gain  =  [(weight at the time of the 50-gram GCT − pre-pregnancy weight)/pre-pregnancy weight] * 100 and was classified into four groups based on increments of 10%.

In the univariate analysis, the association between each TD-FD group and the risk factors for the mean glucose level was analyzed using analysis of variance or Student's t test, as appropriate; and the association between each TD-FD group and the risk factors for the glucose cut-off level of 140 mg/dL was analyzed using a chi-square test. In the multivariate analysis, first, a multiple linear regression model was used to estimate the relationship between each TD-FD group and the glucose levels response to the GCT (continuous variables), adjusted for other risk factors. Second, we used the multiple logistic regression model to further describe the impact of TD-FD on a positive GCT (glucose level ≥140 mg/dL as the event). The significance level was set at 0.05, and all tests were two-tailed. SAS 9.2 software was used for the data analysis (SAS Institute, Cary, NC, USA).

## Results

**Figure 1:**
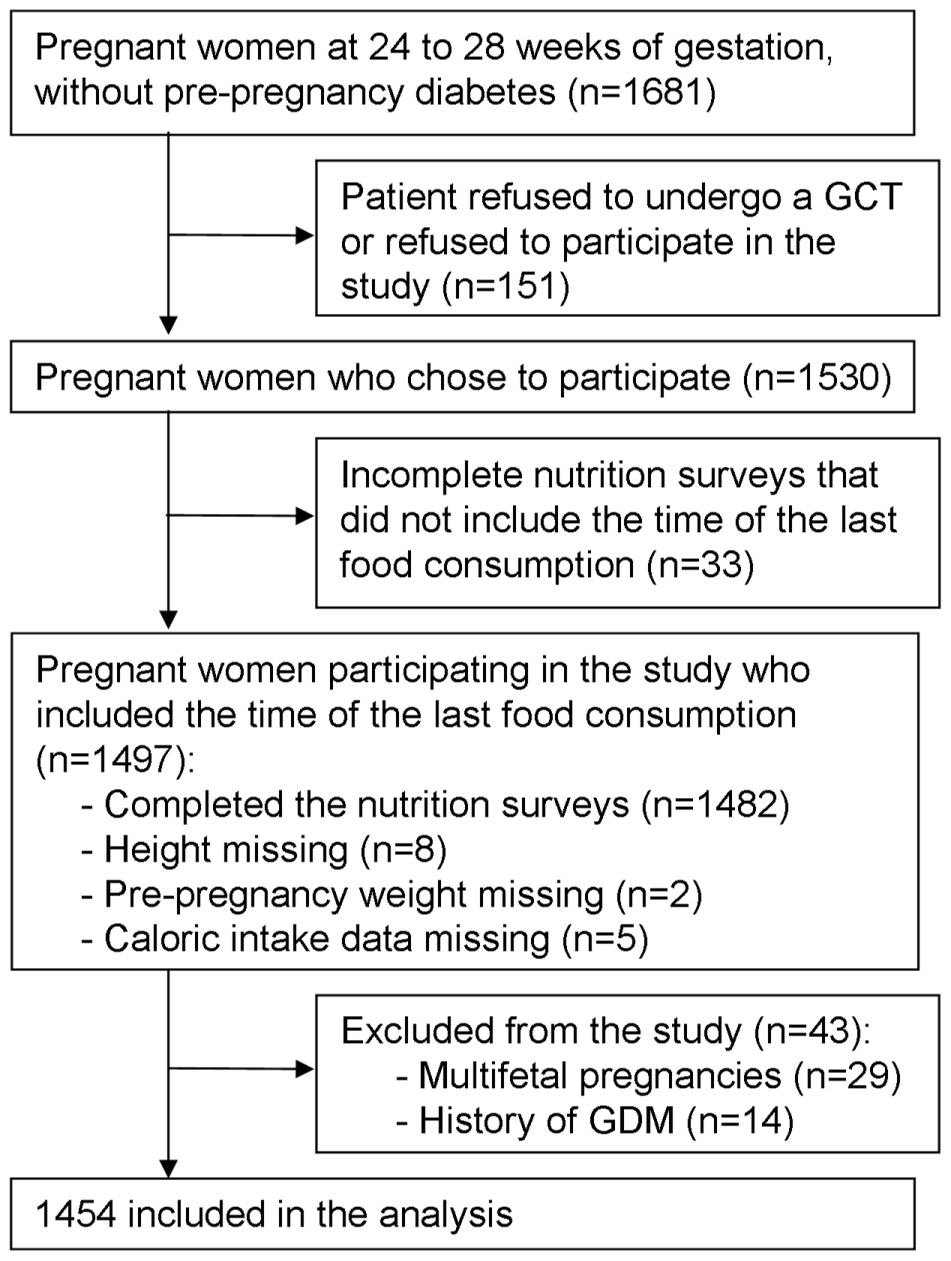
Flowchart of methods.

Of the 1681 patients at 24–28 weeks of gestation without pre-pregnancy diabetes, 91% (n = 1530) underwent a 50-gram GCT and were enrolled in this study. Thirty-three women without information regarding their FD, 29 women with multi-fetal pregnancies, and 14 women with a history of GDM were excluded from the analysis. In total, 1454 women were included in the analysis (**Figure 1**).

**Table 1** shows the distribution of risk factors across the TD-FD groups. These risk factors, with the exception of pre-pregnancy BMI, differed significantly across the TD-FD groups.

**Table 1:** Distribution of risk factors across the TD-FD groups.

Variable	Number	Morning (9 AM–12 AM)						Afternoon (12–5 PM)						Night (5–10 PM)						*P* *
		≤1 hr		1–2 hr		>2 hr		≤1 hr		1–2 hr		>2 hr		≤1 hr		1–2 hr		>2 hr		
Cases No.	1454	151		158		179		124		162		180		140		179		181		
Nulliparous status																				0.01
Yes	737	85	(56.3)	70	(44.3)	83	(46.4)	63	(50.8)	76	(46.9)	83	(46.1)	92	(65.7)	94	(52.5)	91	(50.3)	
No	717	66	(43.7)	88	(55.7)	96	(53.6)	61	(49.2)	86	(53.1)	97	(53.9)	48	(34.3)	85	(47.5)	90	(49.7)	
Maternal age (y)																				
<25	153	14	(9.3)	13	(8.2)	15	(8.4)	21	(16.9)	16	(9.9)	28	(15.6)	12	(8.6)	14	(7.8)	20	(11.1)	0.049
25–30	510	59	(39.1)	48	(30.4)	57	(31.8)	45	(36.3)	53	(32.7)	59	(32.8)	55	(39.3)	70	(39.1)	64	(35.4)	
30–35	564	53	(35.1)	59	(37.3)	75	(41.9)	41	(33.1)	61	(37.7)	68	(37.8)	57	(40.7)	77	(43.0)	73	(40.3)	
≥35	227	25	(16.6)	38	(24.1)	32	(17.9)	17	(13.7)	32	(19.8)	25	(13.9)	16	(11.4)	18	(10.1)	24	(13.3)	
Pre-pregnancy BMI (kg/m^2^)																				
<18.5	221	27	(17.9)	19	(12.0)	25	(14.2)	29	(23.6)	29	(18.0)	29	(16.3)	14	(10.1)	20	(11.2)	29	(16.1)	0.65
18.5–24	925	98	(64.9)	103	(65.2)	111	(63.1)	71	(57.7)	100	(62.1)	117	(65.7)	93	(67.4)	117	(65.4)	115	(63.9)	
24–27	178	18	(11.9)	20	(12.7)	21	(11.9)	15	(12.2)	20	(12.4)	21	(11.8)	17	(12.3)	24	(13.4)	22	(12.2)	
≥27	120	8	(5.3)	16	(10.1)	19	(10.8)	8	(6.5)	12	(7.5)	11	(6.2)	14	(10.1)	18	(10.1)	14	(7.8)	
Missing	10																			
Weight gain (%)†																				
<10	289	18	(11.9)	38	(24.1)	46	(25.7)	23	(18.6)	39	(24.1)	44	(24.6)	17	(12.1)	29	(16.2)	35	(19.4)	0.001
10–20	825	98	(64.9)	78	(49.4)	103	(57.5)	68	(54.8)	77	(47.5)	101	(56.4)	92	(65.7)	109	(60.9)	99	(55.0)	
20–30	291	32	(21.2)	37	(23.4)	28	(15.6)	23	(18.6)	36	(22.2)	29	(16.2)	28	(20.0)	36	(20.1)	42	(23.3)	
≥30	47	3	(2.0)	5	(3.2)	2	(1.1)	10	(8.1)	10	(6.2)	5	(2.8	3	(2.1)	5	(2.8)	4	(2.2)	
Missing	2																			

TD-FD, time of day glucose was measured and fasting duration; GCT, glucose challenge test; BMI, body mass index.

*Data are presented as n (%) and were analyzed using the chi-square test.

† Percentage of gestational weight gain  =  [(weight at time of GCT − pre-pregnancy weight)/pre-pregnancy weight] * 100.

**Table 2** shows the results of the univariate analysis. The mean glucose level did not differ among the TD-FD groups. With the exception of nulliparity, the mean glucose level differed significantly among the risk factors. The results were similar using the continuous and categorical 50-gram GCT glucose levels. There was a trend of increasing glucose level with increasing maternal age and pre-pregnancy BMI. The relationship between glucose level and weight gain showed a J-shape, and the women with a weight gain of 20%–30% had the lowest glucose concentration; the mean±SD was 121.0±27.8 mg/dL, and 63 subjects (21.7%) had a level ≥140 mg/dL.

**Table 2:** The association between TD-FD group and risk factors for the mean glucose level and a cut-off level of 140 mg/dL.

Variable		Number	Glucose level (mg/dL)				
			Mean ± SD	*P* *	≥140 mg/dL, n (%)		*P* †
TD-FD group				0.37			0.39
Morning	≤1 hr	151	124.7±28.3		39	(25.8)	
	1–2 hr	158	124.7±26.8		40	(25.3)	
	>2 hr	179	129.4±29.4		56	(31.3)	
Afternoon	≤1 hr	124	123.5±23.9		25	(20.2)	
	1–2 hr	162	124.6±26.0		35	(21.6)	
	>2 hr	180	126.9±30.1		54	(30.0)	
Night	≤1 hr	140	122.6±31.2		35	(25.0)	
	1–2 hr	179	122.4±26.2		44	(24.6)	
	>2 hr	181	123.8±27.8		50	(27.6)	
Nulliparous status				0.11			0.58
Yes		737	123.7±27.3		187	(25.4)	
No		717	126.0±28.5		191	(26.6)	
Maternal age (y)				<0.001			<0.001
<25		153	115.4±24.5		23	(15.0)	
25–30		510	120.9±25.8		111	(21.8)	
30–35		564	127.0±27.5		165	(29.3)	
≥35		227	134.8±31.9		79	(34.8)	
Pre-pregnancy BMI (kg/m^2^)				<0.001			0.02
<18.5		221	120.6±25.8		49	(22.2)	
18.5–24		925	123.5±27.5		230	(24.9)	
24–27		178	129.6±28.2		55	(30.9)	
≥27		120	136.6±31.0		42	(35.0)	
Missing		10	-				
Weight gain (%)‡				0.004			0.02
<10		289	128.9±31.4		90	(31.1)	
10–20		825	124.5±26.5		208	(25.2)	
20–30		291	121.0±27.8		63	(21.7)	
≥30		47	130.1±25.7		17	(36.2)	
Missing		2	-				

TD-FD, time of day glucose was measured and fasting duration; GCT, glucose challenge test; BMI, body mass index.

*Data were analyzed using analysis of variance or Student's t test as appropriate.

† Data were analyzed using the chi-square test.

‡ Percentage of gestational weight gain  =  [(weight at time of GCT − pre-pregnancy weight)/pre-pregnancy weight] * 100.

**Table 3** shows the results of the multivariate analysis. First, in the multiple linear regression model, after adjusting for the risk factors, the estimate coefficients (β) of the “night, ≤1 hr” and “night, 1–2 hr” TD-FD groups revealed significantly lower glucose concentrations [β (95% confidence interval [CI]): −6.46 (−12.53, −0.38) and −6.85 (−12.50, −1.20)] compared with the “morning, >2 hr” group. Second, for further describing the impact of TD-FD on a positive GCT for potential clinical practice, a multiple logistic regression model was used. The TD-FD groups “afternoon, ≤1 hr” and “afternoon, 1–2 hr” showed significantly lower odds ratios (OR) of a positive GCT compared with the “morning, >2 hr” group; the adjusted ORs (95% CI) were 0.54 (0.31–0.95) and 0.58 (0.35–0.96), respectively.

**Table 3:** Multivariate analysis of the TD-FD groups and the GCT adjusted for risk factors (n = 1444).

Variable		Multiple linear regression			Multiple logistic regression		
		β	95% Confidence interval	*P*	Odds ratio	95% Confidence interval	*P*
Intercept		67.06	(53.04–81.09)	<0.001			
TD-FD group							
Morning	≤1 hr	−4.11	(−10.02, 1.81)	0.17	0.78	(0.48, 1.28)	0.33
	1–2 hr	−5.95	(−11.79, −0.11)	0.046	0.68	(0.42, 1.11)	0.12
	>2 hr	0			1		
Afternoon	≤1 hr	−4.74	(−11.02, 1.54)	0.14	0.54	(0.31, 0.95)	0.03
	1–2 hr	−4.58	(−10.39, 1.24)	0.12	0.58	(0.35, 0.96)	0.03
	>2 hr	−1.61	(−7.26, 4.04)	0.58	0.99	(0.62, 1.56)	0.95
Night	≤1 hr	−6.46	(−12.53, −0.38)	0.04	0.73	(0.44, 1.22)	0.23
	1–2 hr	−6.85	(−12.50, −1.20)	0.02	0.72	(0.45, 1.15)	0.17
	>2 hr	−4.65	(−10.29, 0.99)	0.11	0.88	(0.55, 1.39)	0.57
Nulliparous status							
Yes		1.29	(−1.63, 4.21)	0.38	1.15	(0.90, 1.48)	0.27
No		0			1		
Maternal age (y)		1.28	(0.96, 1.61)	<0.001	1.08	(1.05, 1.11)	<0.001
Pre-pregnancy BMI		1.00	(0.55, 1.45)	<0.001	1.04	(1.00, 1.08)	0.04
Weight gain (%)*							
<10		1.42	(−3.50, 6.34)	0.57	1.25	(0.82, 1.91)	0.30
10–20		0.96	(−2.74, 4.66)	0.61	1.06	(0.76, 1.48)	0.73
20–30		0			1		
≥30		10.39	(1.99, 18.79)	0.02	2.38	(1.22, 4.67)	0.01

TD-FD, time of day glucose was measured and fasting duration; GCT, glucose challenge test; β, regression coefficient; BMI, body mass index.

*Percentage of gestational weight gain  =  [(weight at time of GCT − pre-pregnancy weight)/pre-pregnancy weight] * 100.

## Discussion

Our findings suggest that the TD and FD influence the glucose level following a 1-hour, 50-gram GCT in pregnant women. The patients who were examined in the afternoon and at night without a sufficient fasting interval have had significantly lower glucose measurements in the multivariate analysis. Because the TD and FD are modifiable factors in screening for and diagnosing GDM, they should be considered in clinical practice and epidemiological studies [5], [7], [8], [13]–[15].

We used linear and logistic regression models to determine whether TD and FD were predictors of the glucose concentrations of 50-g GCT and the magnitude of this relation in the current study. The two models both showed the glucose concentrations of 50-g GCT, which performing in the afternoon and night, were lower than that performing in the morning. Although afternoon groups in linear model and the night groups in logistic regression model did not reach the statistical significance, the trends were consistent. We speculated the differences between the two models were caused by the type of outcomes (continuous and binary variables) and the type of results they produced [16].

Additionally, the findings might have implications for the IADPSG guidelines for GDM for both the 2-step approach and the 1-step, 2-hour, 75-gram OGTT. The universal standard for the glucose cut-off value of the 1-hour, 50-gram GCT should be further validated [17], [18]. Although the effects of the TD and FD on the glucose level were mild, these slight changes could affect the diagnosis of GDM at the threshold glucose levels [19]. Our findings indicate that the TD and FD should be considered when screening for and diagnosing GDM in clinical practice. Thus, standardizing the TD and FD for the 1-hour, 50-gram GCT, as well as other glucose tolerance tests, is an important goal.

Maternal glucose metabolism during pregnancy differs from that in non-pregnancy. Previous studies have examined the diurnal glycemic profile and the influence of food intake during pregnancy, using continuous glucose monitoring systems (CGMS) that measure the interstitial glucose levels in subcutaneous tissues every 5 minutes for 72 consecutive hours [20]. The roles of systemic inflammation and insulin resistance in glucose regulation during pregnancy have been investigated [21]. The novel metabolomics approach has recently been used to further explore maternal metabolism during pregnancy [22]. Metabolomics data from 67 mothers of Northern European ancestry from the HAPO study revealed perturbations in the metabolism of major macronutrients and amino acid degradation pathways in mothers with high- versus low-fasting glucose [23]. Further mechanistic studies on the effects of the TD and FD on maternal metabolism during pregnancy are needed.

In addition, we showed that age, pre-pregnancy BMI, and weight gain affect the relationships among the TD, FD and glucose measures. The findings regarding age, maternal obesity, and parity are consistent with previous findings [2], [5]. Our findings support prior evidence that weight gain during pregnancy is an important factor [24]–[27]. Our findings support the recommendation that the TD and FD be standardized in epidemiological studies.

The strength of our study is its detailed assessment, using a relatively large population, of the TD and FD, which are modifiable factors that are important in screening for and diagnosing GDM. The limitations of our study are that other factors might influence the glucose level and that detailed diet records and physical activity data were lacking [28]. Further research is needed to examine the generalizability of our findings to other ethnic groups.

## Conclusions

Our results demonstrate the importance of standardizing the TD and FD for the 1-hour, 50-gram GCT; these factors have previously been ignored in clinical practice and epidemiological investigations. To avoid bias and misclassification when using a glucose tolerance test to screen for and diagnose GDM, the TD and FD should be considered [22].
